# The Evaluation of the Effectiveness of Austrians Disease Management Program in Patients with Type 2 Diabetes Mellitus - A Population-Based Retrospective Cohort Study

**DOI:** 10.1371/journal.pone.0161429

**Published:** 2016-08-17

**Authors:** Regina Riedl, Martin Robausch, Andrea Berghold

**Affiliations:** 1 Institute for Medical Informatics, Statistics and Documentation, Medical University of Graz, Graz, Austria; 2 Controlling Department (ÄIRCON), Lower Austria Health Insurance Fund, St. Pölten, Austria; Universita degli Studi di Perugia, ITALY

## Abstract

**Aim:**

To evaluate the effectiveness of the Austrian Disease Management Program (DMP) ‘Therapie aktiv—Diabetes im Griff’ for patients with type 2 diabetes mellitus concerning patient-relevant outcomes (mortality, myocardial infarction and stroke) and costs.

**Methods:**

Based on routine health insurance data, we conducted a population-based retrospective cohort study using a propensity score (PS) matched control group design. The DMP-group consists of participants enrolled in the program during 2008 and 2009 (n = 7181). Out of 208.532 patients with no participation in the DMP up to 2013, PS-matched controls were selected with a matching ratio 1:3. In the PS-model, patient’s characteristics, form of antidiabetic drug therapy, several prescriptions, the number of hospital admissions and days, main discharge diagnoses and costs at baseline were included.

**Results:**

Over a follow-up period of four years, we observed a significantly lower mortality rate in the DMP-group (9.4%) in comparison with the control group (15.9%, *p*<0.001). The cumulative number of hospital days and mean annual hospital costs were lower for DMP-participants resulting in significantly lower mean annual total costs, amounting to € 8226.80 per patient in the DMP-group and € 9231.10 in the control group respectively (*p*<0.001).

**Conclusions:**

The evaluation shows a survival benefit and an average reduction of costs for participants in the DMP compared with the control-group. Despite we took great effort to ensure comparable groups, we cannot entirely rule out an influence by residual and unmeasured confounding due to the observational study design and the use of routine data. However, the results indicate that the disease management program implemented in Austria improves quality of care for patients with type 2 diabetes mellitus.

## Introduction

In 2014, about 387 million people worldwide, or 8.3% of the adult population aged 20–79 years, are estimated to have diabetes with increasing incidence [1]. In Austria about 573,000–645,000 people suffer from diabetes mellitus, which represents 8%—9% of Austria's population [2]. Diabetes mellitus is associated with serious long-term complications such as cardiovascular disease, blindness, kidney failure, and amputation of the lower extremities, resulting in increased use of medical services, lower quality of life and reduced life expectancy [3]. Moreover, the medical care of diabetic patients leads to high healthcare costs and poses severe challenges for healthcare systems worldwide [4, 5].

To improve the quality of life for diabetic patients, to extend their life time in good health and further to reduce costs, disease management programs (DMPs) for chronic care, including different forms of care coordination and self-management support, were introduced in many countries since the early nineties [6]. In Germany, for example, primary-care based DMPs were implemented nationwide in 2002, including a DMP for type 2 diabetes mellitus [7]. In Austria, the DMP called “Therapie aktiv—Diabetes im Griff” (http://www.therapie-aktiv.at) has the objective to organize long term and high-quality care for patients with type 2 diabetes mellitus. The implementation of this program started in 2007 across most regions of Austria. The participation is voluntary and free of charge for physicians (general practitioners and internists in private practice) and patients. Physicians receive a basic training before they can work as so called “DMP-physicians”. The DMP includes the implementation of evidence-based clinical guidelines and assures that the necessary medical examinations are provided on a regular basis. Patient empowerment is an important component of the program with shared individual target agreements by the patient and the physician. Patients are offered lifestyle advice to enable them to change their diet habits and to encourage physical activity. Regular documentation including information on medical parameters, treatment, target agreements and quality of life is carried out by the DMP-physicians. Currently, about 45.000 diabetic patients are included in the program.

While it is important to monitor the impact of the program for the patients enrolled, the effectiveness of the DMP has to be evaluated compared with a control group [8]. So far, one cluster-randomized controlled trial on the effectiveness of the DMP in Austria was conducted with the primary endpoint change in HbA1c from baseline to one year after enrollment [9]. There were no significant improvements in metabolic control in the intervention group compared with the control group, but the process quality of care was improved. Similar results were observed after two years of follow-up [10]. However, this study was performed only for diabetic patients in one region (Salzburg) with a follow-up of up to two years.

Although a randomized design would be desirable for evaluation, observational study designs might be more suitable especially for long-term effects (i.e. overall mortality, cardiovascular diseases) in population-wide disease management programs frequently implemented in an operational setting. Therefore, studies with longer follow-up periods based on routine data are useful to get more insight in the effectiveness of DMPs.

The aim of this observational study was to evaluate the impact of the DMP in Austria on patient-relevant outcomes (mortality, myocardial infarction and stroke) and costs compared with patients with type 2 diabetes mellitus under routine care.

## Materials and Methods

### Study design and data

To evaluate the DMP we performed a retrospective cohort study using a propensity score (PS) matched control group design with baseline years 2007/2008 and follow-up until 2012/2013. The study was based on routine health insurance data in agreement with the Austrian general social insurance act (LEICON database). Patient information was anonymized and de-identified prior to analysis. In LEICON, the data of 13 major health insurance carriers in Austria are included with coverage of more than 90% of the statutory health insured persons. The identification of patients with type 2 diabetes mellitus in the LEICON database is based on the form of antidiabetic drug therapy, classified according to the Anatomical Therapeutic Chemical (ATC) code. In detail: the prescription of an oral antidiabetic drug (OAD, ATC-code: A10B), a combination therapy of OAD and insulin (ATC-codes: A10B and A10A) or insulin therapy only. For patients receiving insulin therapy only, age restriction is applied (50 years or older) to exclude patients with type 1 diabetes mellitus. Additionally, patients with type 2 diabetes mellitus and no antidiabetic drug therapy are identified by 4 or more blood glucose level measurements or two or more HbA1c measurements.

For this study, patients with type 2 diabetes mellitus had to be registered in LEICON in the baseline years throughout 2012 or deceased. The DMP-group consisted of patients who enrolled in the program between January 1, 2008 and December 31, 2009. To ensure that the patients actively participated in the DMP, they had to have at least one DMP-documentation by their physicians after enrollment. The control group consisted of diabetic patients not enrolled in the DMP before December 31, 2013 and predominantly (more than 80% visits) under treatment of non DMP-physicians. Both groups included only patients still alive on December 31, 2008, for baseline year 2007 and still alive on December 31, 2009, for baseline year 2008.

For the evaluation data concerning patient’s characteristics, prescriptions (S1 Table), number of hospital admissions and days, main diabetes-relevant admission and discharge diagnoses (S2 Table) and costs for in- and outpatient care per calendar year were provided.

### Endpoints

Patient-relevant outcomes and the economic impact were considered. The primary outcome for the medical effectiveness of the DMP was overall mortality. In addition, the diabetes-specific complications myocardial infarction, stroke and stroke/non-traumatic intracranial bleedings identified using the International Classification of Diseases (ICD-10) codes (www.who.int/classifications/icd/en): I21-I22, I63 and I60-I64, respectively, were evaluated. With regard to the economic impact, total costs (including outpatient physician services costs, hospital costs, prescription costs, transportation costs) are defined as the primary outcome. Secondarily, the single cost components and the number of hospital admissions and days were investigated. Outpatient physician services, prescription and transportation costs are based on direct claims data from the health insurance carriers during the investigated time frame. These include all outpatient physician services, except dentistry service, prescriptions reimbursed by health insurance and transportation service. Hospital costs in LEICON are routinely calculated from the individual patient’s length of hospital stays combined with average costs for hospital care, this is due to the central hospital’s yearly lump-sum funding.

### Statistical Analysis

#### Sample size considerations

Sample size considerations were performed for the patient-relevant outcomes and are based on the mortality rate of diabetic patients in Styria in 2006 and the evaluation results from Germany [11, 12]. Under the assumption of a 5-year mortality rate of 18% in the control group and a reduction of 2.7% (15%) in the DMP-group, for a ratio 1:3 3168 DMP-participants and 9599 controls would be needed to achieve a power of 90% for a two-sided chi square test with a significance level of 2.5%. For the secondary patient-relevant outcomes we assumed an annual incidence of 1% and a reduction of 25% for DMP-participants resulting in 5639 DMP-participants and 17087 controls. Based on these considerations, all patients who enrolled in the DMP in the years 2008 and 2009 were included.

#### Propensity score calculation and matching

The PS, defined as probability of participating in the DMP conditional on baseline covariates [13], was calculated using a multivariate logistic regression with DMP-participation as the dependent variable. As independent baseline covariates patient’s characteristics (sex, age, prescription fee (yes/no)), form of antidiabetic drug therapy (none, oral antidiabetic drugs (OAD) only, insulin only, OAD and insulin), several prescriptions (antihypertensive drugs, lipid modifying agents, psychiatric medications and analgesics) (S1 Table), the number of hospital days (none, 1–7, 8–14, 15–30, >30 days), main diabetes-relevant admission and discharge diagnoses (S2 Table) summarized into a single variable (yes, if one or more diagnoses were present, no otherwise) and total costs were included to reflect sociodemographic status and health status at baseline [7, 11, 12, 14]. Based on the estimated PS, for every DMP-participant three controls were matched to increase precision using a nearest-neighbour-matching algorithm without replacement adapted from the SAS macro from Coca-Perraillon [15]. The PS calculation and the matching was performed stratified by the baseline years 2007 and 2008 and stratified by the participating regions of Austria (Burgenland, Lower Austria, Upper Austria, Salzburg, Styria, Vorarlberg and Vienna). To assess the quality of the matching, i.e. if the covariates are balanced between the matched groups, standardized difference between the groups were calculated before and after matching [16]. A standardized difference close to zero indicates good balance of the covariate between the DMP-group and the controls.

#### Analysis

The primary patient-relevant outcome, mortality, was analyzed using Kaplan-Meier and a Cox-Proportional Hazard Model. To account for the matched nature of the data, a robust sandwich estimator was used [17]. An observation was censored if the patient was still alive after 4 years of follow-up. The observational periods for the DMP-participants and their matched controls are: January 1, 2009 to December 31, 2012 and January 1, 2010 to December 31, 2013 for the two baseline years 2007 and 2008, respectively. The results are presented as hazard ratio (HR) with a 95% confidence interval (CI). For cost analysis, the mean annual total costs per person over the 4 year follow-up period 2009/2010–2012/2013 are calculated and compared between the DMP-participants and controls using a GEE-Modell with gamma-distribution and log-link accounting for the matching [18]. For the GEE-Model, PROC GENMOD was used assuming an exchangeable correlation structure. Additionally, a 95% CI for the mean annual total cost differences between the groups was calculated using bootstrap-methods [19, 20]. This was done by drawing (with replacement) 10000 repeated random samples of the same size as the original sample. To account for the matching, we draw the bootstrap samples from the matched sets as recommended in [20]. Secondary outcomes (diabetes-specific complications, single cost components, the number of hospital admissions and days) were analyzed descriptively. Furthermore, sensitivity analyses were performed to investigate changes in the outcome parameters under varying inclusion/exclusion criteria. For the two primary endpoints a two-sided significance level of 2.5% was used to indicate statistical significance. Matching and statistical analysis were performed using SAS Version 9.2.

## Results

**Fig 1:**
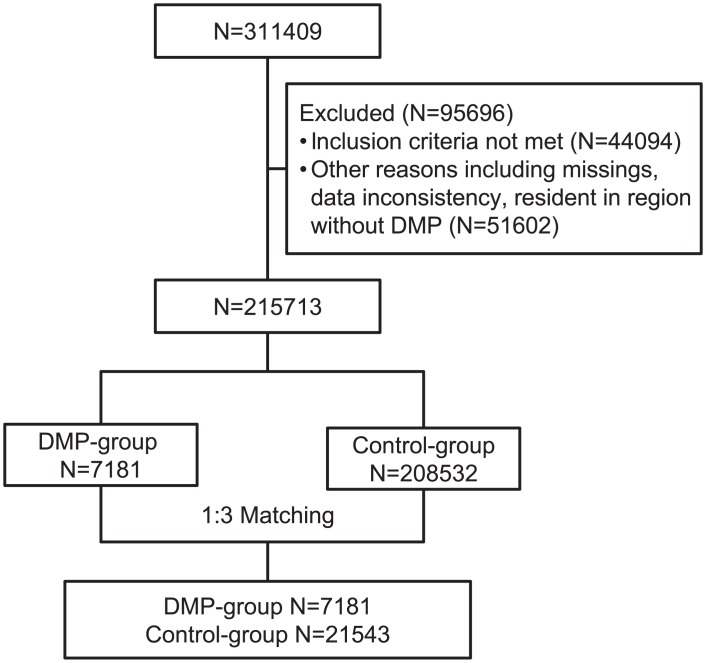
Flowchart of type 2 diabetic patients considered for evaluation study. Inclusion/Exclusion criteria: DMP-group: enrollment in DMP between January 1, 2008 and December 31, 2009; at least one DMP-documentation. Control group: no enrollment in DMP before December 31, 2013; predominantly under treatment of non DMP-physicians. Both groups: patients registered in LEICON database in the baseline years throughout 2012 or deceased; still alive on December 31, 2008, (baseline year 2007) and December 31, 2009, (baseline year 2008).

A total of 311409 patients were registered in LEICON in the baseline years 2007 and 2008 up to 2012. From those, 44094 (14.2%) patients did not meet the inclusion criteria and 51602 (16.6%) were excluded due to missing values, data inconsistency and residency in a region without DMP in the baseline years 2007 or 2008. On the remaining 215713 patients (7181 DMP-group, 208532 control group), a propensity score matching with 1:3 ratio was performed yielding 7181 DMP-participants and 21543 controls for analysis (Fig 1).

### Balance

**Fig 2:**
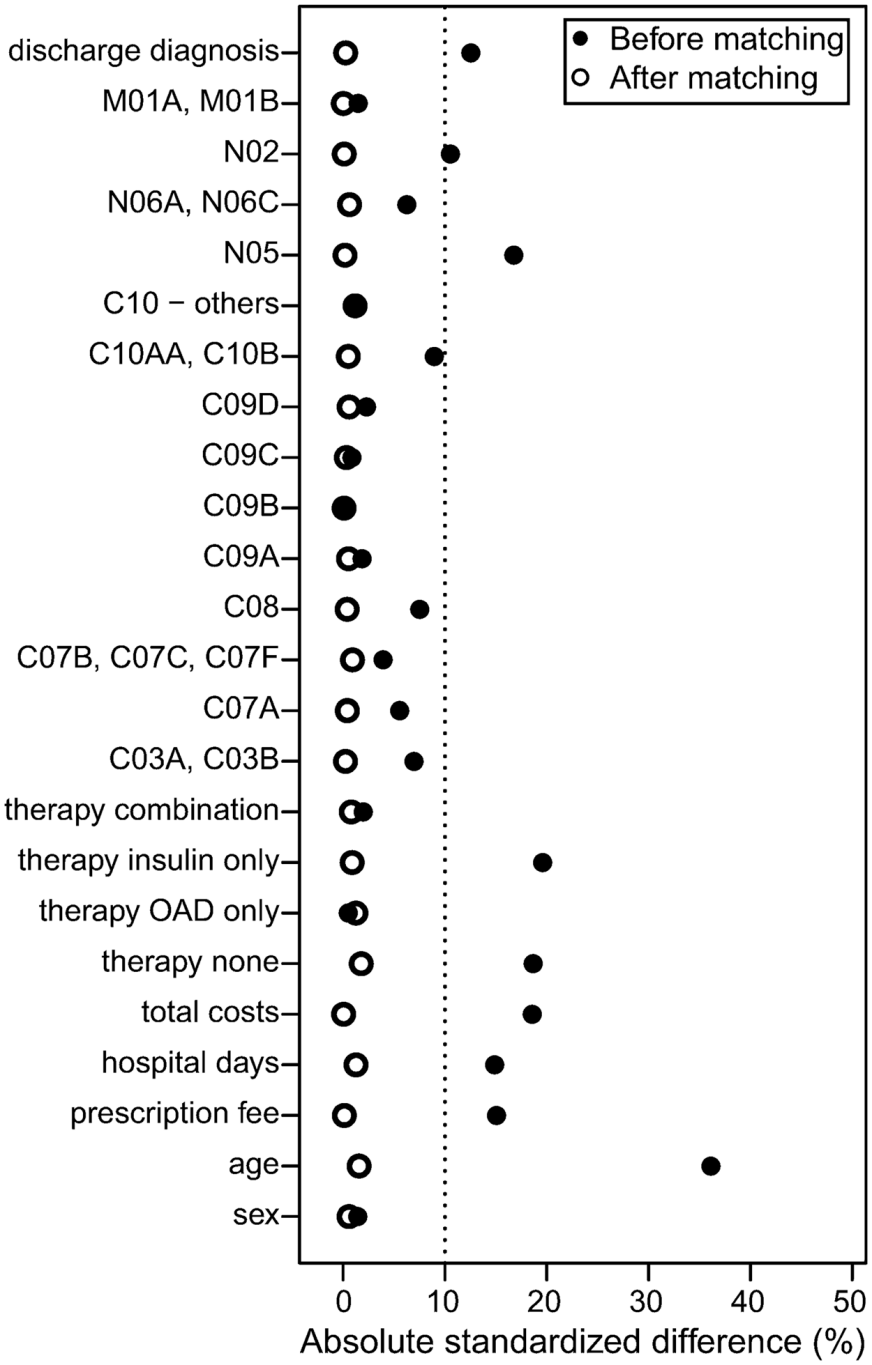
Absolute standardized differences (%) between DMP-participants and controls before and after matching. Therapeutic subgroups of the Anatomical Therapeutic Chemical (ATC) codes: Analgetic medication: N02, M01A, M01B; Psychiatric medication: N05, N06A, N06C; Lipid modifying agents: C10AA, C10B, C10AB, C10AC, C10AD, C10AX; Antihypertensive drugs: C03A, C03B, C07A, C07B, C07C, C07F, C08, C09A, C09B, C09C, C09D.

Before matching large imbalances were observed for age, the form of antidiabetic drug therapy, total costs and hospital days. After matching all measured baseline characteristics were similar between the groups (Fig 2, S3 Table). In the matched data, 50.9% of the patients were female and the mean age was 64.2 (±11.4) years. At baseline, 66.5% of the patients received oral antidiabetic drugs (OAD) only, 6.7% insulin only, 10.6% both and 15.9% diabetic patients received no antidiabetic drug therapy.

### Patient-relevant outcomes

**Fig 3:**
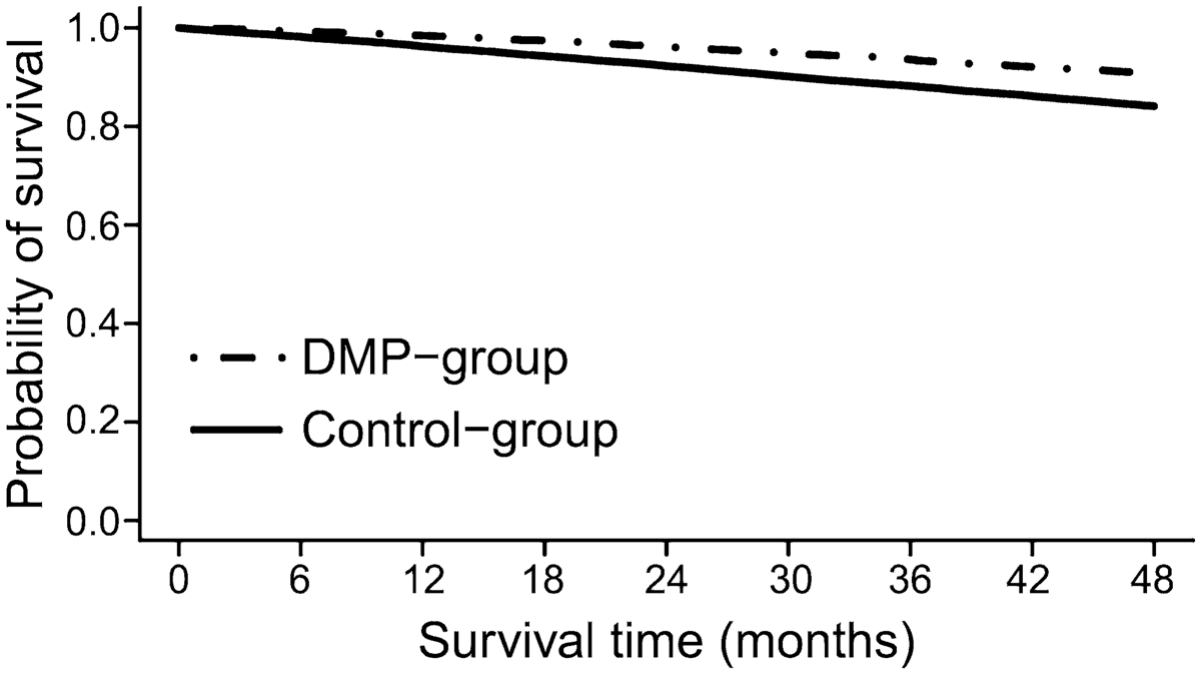
Kaplan-Meier curves for DMP-participants and controls.

A comparison of the patient-relevant outcomes between the matched groups is presented in Table 1. Within 4 years after DMP enrollment, 9.4% (674/7181) of the patients died in the DMP-group, whereas in the control-group 15.9% (3426/21543) of the patients died. Mortality in the DMP-group was significantly reduced compared with the control-group (HR = 0.57, 95% CI: 0.52–0.61, *p*<0.001). In Fig 3 the Kaplan-Meier curves are shown. For the secondary outcomes, slightly lower percentages were observed for the DMP-participants (myocardial infarction: 2.0% (143/7161), stroke 2.2% (159/7161)) compared with controls (myocardial infarction: 2.3% (485/21044), stroke 2.6% (542/21044)). The percentage of at least one of the investigated diabetic-specific complications was 5.0% (359/7161) in the DMP-group and 6.1% (1279/21044) in the control-group.

**Table 1:** Comparison of patient-relevant outcomes between the DMP-group and the control-group for a follow-up period of 4 years.

	DMP-group N = 7181		Control-group N = 21543	
	N	%	N	%
Mortality	674	9.39	3426	15.90
HR (95% CI)	0.57 (0.52–0.61)			
Diabetes-specific complications^a^				
Myocardial infarction (ICD: I21, I22)	143	2.00	485	2.30
Stroke/non-traumatic intracranial bleedings (ICD: I60-I64)	225	3.14	828	3.93
Stroke (ICD: I63)	159	2.22	542	2.58
Any complication ^b^	359	5.01	1279	6.08

^a^ N = 7161 in the DMP-group and N = 21044 in the control-group due to missing values

^b^ Included ICD: I21-I22 and/or I60-I64

### Economic impact

The average annual total costs per person over the 4-year period 2009/2010–2012/2013 amounted € 8226.80 in the DMP-group and € 9231.10 in the control-group (*p*<0.001). The cost difference calculated by bootstrap was € 1031.30 (95% CI: € 685.00—€ 1361.40). The mean annual cost components were similar between the groups, except for hospital costs and costs for outpatient physician services. Slightly higher outpatient physician services costs and lower hospital costs were observed in the DMP-group. The cumulative number of hospital days over 4 years follow-up tended to be lower for DMP-participants (median 16 days) compared with controls (median 18 days). There was no difference in the cumulative number of hospital admissions between the groups (median 3 in both groups) (Table 2).

**Table 2:** Comparison of the economic impact between the DMP-group and the control-group for a follow-up period of 4 years.

	DMP-group N = 7161	Control-group N = 21044
Mean total costs per year	8226.80€	9231.10€
Outpatient physician services costs	718.80€	654.40€
Hospital costs	6196.60€	7165.40€
Prescription costs	1243.10€	1296.50€
Transportation costs	68.30€	114.80€
Hospital admissions and days		
Hospital admissions and days >0, N (%)	5201 (72.6)	14944 (71.0)
Cumulative number of hospital days >0 (mean/median)	29.5/16	32.5/18
Cumulative number of hospital admissions >0 (mean/median)	4.1/3	4.3/3

## Discussion

In this population based cohort study, we evaluated the impact of the DMP in Austria for patients with type 2 diabetes mellitus on patient-relevant and economic outcomes in DMP-participants compared with propensity score matched controls. As primary endpoints mortality and total costs were considered. Both endpoints showed an association between participation in the DMP, and a reduction in mortality and total costs, respectively. Over a follow-up period of four years, the mortality rate in the DMP-group was 9.4% and in the control-group 15.9%. Likewise, the average annual total costs in the DMP-group were reduced on average by about € 1000.

In Germany and Austria the DMP is comparable, the main difference being the implementation of the program; nationwide implementation in Germany and decentralized in Austria [21]. In evaluation studies of the German DMP, similar results were observed which are summarized in a recent systematic review [22]. All studies refer to the first years of DMP implementation, do not go beyond the year 2008 and are very heterogeneous in terms of design, scale and considered endpoints. In summary, positive effects resulted in the evaluation studies in Germany on mortality and improved results concerning process parameters; however, a mixed picture in other investigated parameters like costs is shown. The BARMER study [12, 23] is most comparable to our study, with the exception that no patients without antidiabetic drug therapy were considered and the methodology for the selection of the control-group at both, the parameters included in the PS calculation as well as the matching method used differs in detail. Thus, the demographics slightly differ, for example, the participants in the BARMER study were on average 67 years old, in our study 64 years. After a follow-up period of 3 years, Drabik et al [12] observed a somewhat higher reduction in mortality of 7.5% (mortality rate 7.2% in the DMP-group and 14.7% in the control-group) compared with our results, i.e. reduction in mortality of 6.5% after 4 years. Diabetes-specific complications such as myocardial infarction and stroke, descriptively reported in the BARMER study, were lower in the DMP-group compared to controls [23]. After 4 years follow-up, we observed only a small difference in diabetes-specific complications between the groups (any complication in DMP-group 5.0%: controls: 6.1%). This stands in contrast to our observed substantial reduction in all-cause mortality. In other long-term evaluations of management and self-management programs for diabetic patients a decrease in mortality and cardiovascular events were observed [24, 25]. Our observed modest differences in diabetes-specific complications might be based on the fact that just the admission diagnosis and two discharge diagnoses are documented in the LEICON database and we probably missed some cases. However, in a study from Linder et al [14], no differences between the DMP-participants and controls were observed for stroke and myocardial infarction after 2 years follow-up. In Austria, Ostermann et al [26] observed an improved quality of outpatient care and lower hospitalization for DMP-participants compared with controls in 2009. This is in agreement with our observations that DMP-participants tended to have shorter hospital stays. After one year follow-up, improvements in metabolic control (although not significant), weight loss, cholesterol level and process quality were observed in Sönnichsen et al [9]. Overall, this might contribute to a lower mortality rate for DMP-participants as observed in our study.

The fewer deaths and shorter hospital stays in the DMP-group are reflected in our observed lower total costs which mostly consist of costs for inpatient hospital care. Likewise, the slightly higher outpatient physician services costs in the DMP-group are indicating that patients enrolled in the program might receive more outpatient/ambulatory health care and use more diabetes relevant health services from general practitioners, internists or ophthalmologists compared to non-participants [26]. Generally, there are only small differences in the single cost components between the groups, with the exception of hospital costs. Qualitatively similar results were observed in other studies [12, 14, 23].

For evaluation studies on the effectiveness of DMPs in other countries it must be noted that both the health systems and the DMPs are organized differently. Additionally, the studies are widely heterogeneous in terms of design, choice of outcome measure and observational period. An overview of approaches for chronic disease management and evaluations in Europe is given in Nolte et al [6]. Examples of implemented multidisciplinary management and self-management programs in non-European countries are given in [24, 25, 27, 28, 29, 30].

Frequently investigated parameters to assess the effectiveness of chronic care management programs for diabetes are intermediate clinical outcome measures such as HbA1c and systolic blood pressure. Results from recent meta-analyses generally suggest a positive impact of these programs; however, only moderate improvements in glycaemic control were observed [31, 32].

Our study has several limitations: the main limitation is that this study has no randomized design. Although our PS matching resulted in good balance, this approach adjusts only for measured confounders and further depends on the availability and quality of the routine data. The opportunities and limitations of using routine data for evaluations are discussed in [33]. In our study, the identification of patients with type 2 diabetes mellitus by ICD-10 codes was not possible, since in the outpatient sector in Austria, no standardized coding with ICD-10 is performed. However, ATC codes for prescriptions of pharmaceutical products are well documented and work has been performed to determine the reliability for predicting the ICD code from the ATC code [34]. Additionally, in 2012, the implemented algorithm in the LEICON database was tested for its accuracy to identify patients with type 2 diabetes mellitus. The results showed high sensitivity and specificity of over 80%. However, we cannot completely rule out that misclassification of type 2 diabetes mellitus in the control group is present. Furthermore, we did not have access to clinical data including HbA1c measurements or duration of diabetes which is associated with an increased risk of micro- and macrovascular complications and death [35]. However, to reduce the impact of confounding by disease severity, a large number of diabetes relevant prescriptions and discharge diagnoses were included in the PS calculation. Although, after matching no imbalances were observed in those variables, we cannot rule out that there are still differences between the groups at baseline in terms of other variables (e.g. education, health awareness), which could not be considered due to the secondary use of routine data of the Austrian social insurance institutions. Additionally, our evaluation might be influenced by spill-over effects [36], and by higher motivation of DMP-physicians. The motivation of DMP-physicians is not considered in the matching as only patient characteristics were available. To avoid treatment cross-over in the control-group, we decided to include only control patients which are predominantly under treatment of non DMP-physicians. In a sensitivity analysis without this criterion, no substantial differences in the results were observed (S4 Table). Therefore, our findings may partially be explained by a higher motivation of the patients and the physicians and a less advanced disease in DMP-participants compared to non-participants [37]. A further limitation is that indirect costs including for example additional costs for inability to work could not be considered in our analysis due to limited data availability.

Strengths of the study are the large population based cohort design with follow-up period of 4 years after program enrollment, a broad consideration of matching variables to account for selection bias and the inclusion of patients with type 2 diabetes mellitus and without antidiabetic drug therapy. Including diabetic patients based on drug prescriptions only might reduce the generalizability of the results. In our data, 15.9% (9.5% before matching) belong to diabetic patients without antidiabetic drug therapy. However, in sensitivity analyses without this group we did not observe substantial changes in the results (S5 Table). Additionally, our analysis also includes patients without continuous DMP participation to account for probably less motivated patients. Our inclusion of patients without antidiabetic drug therapy and patients with lesser program activity reflects the effectiveness of the DMP in a realistic way [8].

## Conclusions

In summary, over a follow-up period of four years, we observed a significantly lower mortality rate and a reduction in total costs for the DMP-participants in comparison with the control-group. However, we cannot rule out that our findings might be influenced by confounders like disease severity, education or different health awareness among the comparison groups at baseline. Despite these limitations, our study results indicate that the DMP "Therapie aktiv" improves the care of patients with type 2 diabetes mellitus. Finally, it should also be mentioned that our results relate to the initial phase of the program. Further evaluations of the DMP are important to investigate whether the observed benefits maintain or change over time.

## Supporting Information

S1 Table List of included prescriptions based on Anatomical Therapeutic Chemical (ATC) Classification System. (DOCX)

S2 Table List of included discharge diagnoses based on International Classification of Diseases (ICD10) codes. (DOCX)

S3 Table Descriptive statistics for the DMP-group and the control-group before and after matching. (DOCX)

S4 Table Results for sensitivity analysis including control patients who are predominantly under treatment of DMP physicians. (DOCX)

S5 Table Results for sensitivity analysis excluding type 2 diabetes mellitus patients without antidiabetic drug therapy. (DOCX)
