# Supplementary material for: The Evaluation of the Effectiveness of Austrians Disease Management Program in Patients with Type 2 Diabetes Mellitus - A Population-Based Retrospective Cohort Study
Source: PLoS One. 2016 Aug 17;11(8):e0161429. doi: 10.1371/journal.pone.0161429 (PMC4988720; doi:10.1371/journal.pone.0161429)
Supplement: S1 Table — (DOCX) [file pone.0161429.s001.docx]

**Additional file 1:** List of included prescriptions based on Anatomical Therapeutic Chemical (ATC) Classification System.

|  | **ATC-Codes** |
| --- | --- |
| **Drugs used in diabetes** | |
| Blood glucose lowering drugs, excluding insulins | A10B |
| Insulins and analogues | A10A |
| **Antihypertensive drugs** | |
| Diuretics | C03A, C03B |
| Beta blocking agents (plain) | C07A |
| Beta blocking agents (combinations) | C07B, C07C, C07F |
| Calcium channel blockers | C08 (excluding C08CA06) |
| ACE inhibitors (plain) | C09A |
| ACE inhibitors (combinations) | C09B |
| Angiotensin II antagonists (plain) | C09C |
| Angiotensin II antagonists (combinations) | C09D |
| Renin inhibitors | C09X |
| **Lipid modifying agents** | |
| HMG-CoA reductase inhibitors | C10AA |
| Lipid modifying agents (combinations) | C10B |
| Other lipid modifying agents | C10AB, C10AC, C10AD, C10AX |
| **Psychiatric medication** | |
| Psycholeptics | N05 |
| Psychoanaleptics | N06A, N06C |
| **Analgetic medication** | |
| Analgesics | N02 |
| Anti-inflammatory and antirheumatic products | M01A, M01B |
