# Supplementary material for: The Evaluation of the Effectiveness of Austrians Disease Management Program in Patients with Type 2 Diabetes Mellitus - A Population-Based Retrospective Cohort Study
Source: PLoS One. 2016 Aug 17;11(8):e0161429. doi: 10.1371/journal.pone.0161429 (PMC4988720; doi:10.1371/journal.pone.0161429)
Supplement: S2 Table — (DOCX) [file pone.0161429.s002.docx]

**Additional file 2:** List of included discharge diagnoses based on International Classification of Diseases (ICD10) codes.

| **ICD10-codes** | **Description** |
| --- | --- |
| E10-E14 | Diabetes mellitus |
| E66 | Obesity |
| G62 | Other polyneuropathies |
| G63 | Polyneuropathy in diseases classified elsewhere |
| H30-H36 | Disorders of choroid and retina |
| H43 | Disorders of vitreous body |
| I10-I15 | Hypertensive diseases |
| I20-I25 | Ischemic heart diseases |
| I42-I52 | Other forms of heart disease |
| I61-I69 | Cerebrovascular diseases (without I60) |
| I70-I79 | Diseases of arteries, arterioles and capillaries |
| N08 | Glomerular disorders in diseases classified elsewhere |
| N17-N19 | Renal failure |
| R02 | Gangrene, not elsewhere classified |
| T05.3-T05.5 | Traumatic amputations (feet/legs) |
| T13.6 | Traumatic amputation of lower limb, level unspecified |
