# Supplementary material for: The Evaluation of the Effectiveness of Austrians Disease Management Program in Patients with Type 2 Diabetes Mellitus - A Population-Based Retrospective Cohort Study
Source: PLoS One. 2016 Aug 17;11(8):e0161429. doi: 10.1371/journal.pone.0161429 (PMC4988720; doi:10.1371/journal.pone.0161429)
Supplement: S3 Table — (DOCX) [file pone.0161429.s003.docx]

**Additional file 3:** Descriptive statistics for the DMP-group and the control-group before and after matching.

| **Matching parameters** | **DMP-group**  **N=7181** | | **Control-group** | | | |
| --- | --- | --- | --- | --- | --- | --- |
|  |  |  | **before matching** | | **after matching** | |
|  |  |  | **N=367992**^a^ | | **N=21543** | |
|  | **N**  **mean (SD)** | **%**  **median**  **(min-max)** | **N**  **mean (SD)** | **%**  **median**  **(min-max))** | **N**  **mean (SD)** | **%**  **median**  **(min-max)** |
| sex |  |  |  |  |  |  |
| female | 3672 | 51.1 | 185525 | 50.4 | 10953 | 50.8 |
| male | 3509 | 48.9 | 182467 | 49.6 | 10590 | 49.2 |
| age | 64.0 (10.9) | 65 (18-95) | 68.1 (11.5) | 68 (18-99) | 64.2 (11.6) | 65 (18-99) |
| prescription fee | 2233 | 31.1 | 140730 | 38.2 | 6711 | 31.2 |
| hospital days |  |  |  |  |  |  |
| none | 4882 | 68.0 | 231651 | 63.0 | 14633 | 67.9 |
| 1-7 days | 1109 | 15.4 | 53566 | 14.6 | 3314 | 15.4 |
| 8-14 days | 531 | 7.4 | 32646 | 8.9 | 1619 | 7.5 |
| 15-30 days | 433 | 6.0 | 29467 | 8.0 | 1300 | 6.0 |
| >30 days | 226 | 3.1 | 20662 | 5.6 | 677 | 3.1 |
| hospital days >0 | 13.5 (16.2) | 8 (1-154) | 17.0 (21.1) | 10 (1-743) | 12.9 (14.7) | 8 (1-198) |
| total costs | 2744  (3654) | 1603  (8-63888) | 3567 (5090) | 1888  (4-368969) | 2746  (3517) | 1598  (7-55420) |
| therapy form |  |  |  |  |  |  |
| none | 1112 | 15.5 | 34418 | 9.4 | 3462 | 16.1 |
| OAD only | 4810 | 67.0 | 245604 | 66.7 | 14302 | 66.4 |
| Insulin only | 484 | 6.7 | 45963 | 12.5 | 1508 | 7.0 |
| combination | 775 | 10.8 | 42007 | 11.4 | 2271 | 10.5 |
| prescriptions |  |  |  |  |  |  |
| C03A, C03B | 414 | 5.8 | 27577 | 7.5 | 1229 | 5.7 |
| C07A | 2596 | 36.2 | 142914 | 38.8 | 7832 | 36.4 |
| C07B, C07C, C07F | 643 | 9.0 | 28935 | 7.9 | 1874 | 8.7 |
| C08 (except C08CA06) | 1727 | 24.0 | 100584 | 27.3 | 5219 | 24.2 |
| C09A | 2169 | 30.2 | 114316 | 31.1 | 6454 | 30.0 |
| C09B | 2113 | 29.4 | 107668 | 29.3 | 6349 | 29.5 |
| C09C | 821 | 11.4 | 41058 | 11.2 | 2485 | 11.5 |
| C09D | 1044 | 14.5 | 50565 | 13.7 | 3088 | 14.3 |
| C10AA, C10B | 3531 | 49.2 | 164544 | 44.7 | 10538 | 48.9 |
| C10-others | 418 | 5.8 | 20628 | 5.6 | 1313 | 6.1 |
| N05 | 678 | 9.4 | 54838 | 14.9 | 2047 | 9.5 |
| N06A, N06C | 1389 | 19.3 | 80474 | 21.9 | 4222 | 19.6 |
| N02 | 1095 | 15.2 | 70729 | 19.2 | 3276 | 15.2 |
| M01A, M01B | 3371 | 46.9 | 170034 | 46.2 | 10111 | 46.9 |
| discharge diagnosis total | 877 | 12.2 | 61116 | 16.6 | 2612 | 12.1 |
| discharge diagnosis |  |  |  |  |  |  |
| E10-E14 | 332 | 4.6 | 23044 | 6.3 | 997 | 4.6 |
| E66 | 12 | 0.2 | 361 | 0.1 | 25 | 0.1 |
| G63 | 3 | 0.0 | 357 | 0.1 | 17 | 0.1 |
| G62 | 1 | 0.0 | 176 | 0.0 | 5 | 0.0 |
| H30-H36 | 61 | 0.8 | 4111 | 1.1 | 189 | 0.9 |
| H43 | 4 | 0.1 | 397 | 0.1 | 11 | 0.1 |
| I10-I15 | 110 | 1.5 | 7635 | 2.1 | 317 | 1.5 |
| I20-I25 | 220 | 3.1 | 12686 | 3.4 | 616 | 2.9 |
| I42-I52 | 145 | 2.0 | 11389 | 3.1 | 408 | 1.9 |
| I61-I69 | 68 | 0.9 | 5966 | 1.6 | 224 | 1.0 |
| I70-I79 | 71 | 1.0 | 5599 | 1.5 | 219 | 1.0 |
| N08 | 1 | 0.0 | 47 | 0.0 | 2 | 0.0 |
| N17-N19 | 13 | 0.2 | 2594 | 0.7 | 87 | 0.4 |
| R02 | 3 | 0.0 | 415 | 0.1 | 11 | 0.1 |
| T05.3-T05.5 | 0 | 0.0 | 2 | 0.0 | 0 | 0.0 |
| T13.6 | 0 | 0.0 | 3 | 0.0 | 0 | 0.0 |
| hospital admissions |  |  |  |  |  |  |
| 0x | 4882 | 68.0 | 231651 | 63.0 | 14633 | 67.9 |
| 1x | 1328 | 18.5 | 71433 | 19.4 | 4048 | 18.8 |
| 2x | 550 | 7.7 | 31921 | 8.7 | 1591 | 7.4 |
| >2x | 421 | 5.9 | 32987 | 9.0 | 1271 | 5.9 |
| hospital admissions >0 | 1.8 (1.5) | 1 (1-16) | 2.1 (2.3) | 1 (1-111) | 1.9 (1.9) | 1 (1-41) |

^a^ includes N = 178440 patients with baseline year 2007 and N = 189552 patients with baseline year 2008.
