# Supplementary material for: The Evaluation of the Effectiveness of Austrians Disease Management Program in Patients with Type 2 Diabetes Mellitus - A Population-Based Retrospective Cohort Study
Source: PLoS One. 2016 Aug 17;11(8):e0161429. doi: 10.1371/journal.pone.0161429 (PMC4988720; doi:10.1371/journal.pone.0161429)
Supplement: S4 Table — (DOCX) [file pone.0161429.s004.docx]

**Additional file 4** Results for sensitivity analysis including control patients who are predominantly under treatment of DMP physicians.

|  | **DMP-group**  **N=7181** | | **Control-group**  **N=21543** | |
| --- | --- | --- | --- | --- |
| **Patient-relevant outcomes** | | | | |
|  | **N** | **%** | **N** | **%** |
| Mortality | 674 | 9.39 | 3440 | 16.00 |
| HR (95% CI) | 0.56 (0.52-0.61) | | | |
| Diabetes-specific complications^a^ | | | | |
| Myocardial infarction (ICD: I21, I22) | 143 | 2.00 | 473 | 2.25 |
| Stroke/non-traumatic intracranial bleedings (ICD: I60-I64) | 225 | 3.14 | 771 | 3.66 |
| Stroke (ICD: I63) | 159 | 2.22 | 480 | 2.28 |
| Any complication ^b^ | 359 | 5.01 | 1212 | 5.76 |
| **Economic impact ^a^** | | | | |
| Mean total costs per year | 8226.80€ | | 9238.10€ | |
| Outpatient physician services costs | 718.80€ | | 646.90€ | |
| Hospital costs | 6196.60€ | | 7165.90€ | |
| Prescription costs | 1243.10€ | | 1320.00€ | |
| Transportation costs | 68.30€ | | 105.40€ | |
| Hospital admissions and days |  | |  | |
| Hospital admissions and days 0, N (%) | 1960 (27.4) | | 5122 (28.5) | |
| Hospital admissions and days >0, N (%) | 5201 (72.6) | | 15078 (71.6) | |
| Cumulative number of hospital days >0 (mean/median) | 29.5/16 | | 32.0/18 | |
| Cumulative number of hospital admissions >0 (mean/median) | 4.1/3 | | 4.2/3 | |

^a^ N=7161 in the DMP-group and N=21048 in the control-group due to missing values

^b^ Included ICD: I21-I22 and/or I60-I64
