# Supplementary material for: The Evaluation of the Effectiveness of Austrians Disease Management Program in Patients with Type 2 Diabetes Mellitus - A Population-Based Retrospective Cohort Study
Source: PLoS One. 2016 Aug 17;11(8):e0161429. doi: 10.1371/journal.pone.0161429 (PMC4988720; doi:10.1371/journal.pone.0161429)
Supplement: S5 Table — (DOCX) [file pone.0161429.s005.docx]

**Additional file 5** Results for sensitivity analysis excluding type 2 diabetes mellitus patients without antidiabetic drug therapy.

|  | **DMP-group**  **N=6069** | | **Control-group**  **N=18207** | |
| --- | --- | --- | --- | --- |
| **Patient-relevant outcomes** | | | | |
|  | **N** | **%** | **N** | **%** |
| Mortality | 606 | 9.99 | 2745 | 15.08 |
| HR (95% CI) | 0.64 (0.59-0.70) | | | |
| Diabetes-specific complications^a^ | | | | |
| Myocardial infarction (ICD: I21, I22) | 134 | 2.21 | 450 | 2.50 |
| Stroke/non-traumatic intracranial bleedings (ICD: I60-I64) | 200 | 3.30 | 746 | 4.15 |
| Stroke (ICD: I63) | 141 | 2.33 | 470 | 2.61 |
| Any complication ^b^ | 325 | 5.37 | 1163 | 6.46 |
| **Economic impact ^a^** | | | | |
| Mean total costs per year | 8705.40€ | | 9426.20€ | |
| Outpatient physician services costs | 726.00€ | | 601.80€ | |
| Hospital costs | 6590.10€ | | 7380.50€ | |
| Prescription costs | 1316.30€ | | 1331.10€ | |
| Transportation costs | 72.90€ | | 112.80€ | |
| Hospital admissions and days |  | |  | |
| Hospital admissions and days 0, N (%) | 1601 (26.4) | | 5122 (28.5) | |
| Hospital admissions and days >0, N (%) | 4456 (73.6) | | 12868 (71.5) | |
| Cumulative number of hospital days >0 (mean/median) | 30.8/17 | | 33.7/19 | |
| Cumulative number of hospital admissions >0 (mean/median) | 4.3/3 | | 4.4/3 | |

^a^ N=6057 in the DMP-group and N=17990 in the control-group due to missing values

^b^ Included ICD: I21-I22 and/or I60-I64
